# Effect of Instructing Care Program Through Group Discussion on the Quality of Life of the Parents of the Children Afflicted With Leukemia

**DOI:** 10.5539/gjhs.v8n5p197

**Published:** 2015-10-20

**Authors:** Fariba Asadi Noughabi, Daryoush Iranpoor, Hadi Yousefi, Hakimeh Abrakht, Fatemeh Ghani Dehkordi

**Affiliations:** 1 School of Nursing & Midwifery, Hormozgan University of Medical Sciences, Bandar Abbas, Iran; 2 Internal medicine Department, Bushehr University of Medical Sciences, Bushehr, Iran; 3 School of Nursing & Midwifery, Hormozgan University of Medical Sciences, Bandar Abbas, Iran; 4 Bushehr University of Medical Sciences, Bushehr, Iran; 5 Department of operating Room, Faculty of Paramedicine, Bushehr University of Medical Sciences, Bushehr, Iran

**Keywords:** leukemia, quality of life, Education, children, parents

## Abstract

**Purpose:**

Children long-term involvement with cancer may have a negative impact on the quality of life their parents. Design and implementation of training programs for parents whose children have been diagnosed with leukemia, as the primary caregivers of children, will have a special significance and can contribute to better taking care of such children. The main purpose of the present study was to examine the impact of conducting group discussion, as care program training, on the quality of life parents whose children were suffering from leukemia.

**Methods:**

This quasi-experimental before-after intervention study encompassed two groups of parents (in total 41) of leukemia children. To collect data, a demographic questionnaire and the shortened version of SF-36 questionnaire were used to determine the quality of life of parents. Both groups completed the quality of life questionnaires before and two months after the intervention.

**Results:**

Comparison of the parents’ quality of life mean scores, obtained before and two months after training, showed that promotion in 6 domains of bodily pain, general health, emotional health, role limitation due to emotional problems, social functioning, and vitality were occurred. (P <0.05)

**Conclusions:**

Considering the important role of parents in taking care of children suffering from leukemia, introduction of care program training can be a positive step to help these parents and empower them to manage their children’s problems more systematically and will ultimately lead to improved quality of life of parents.

## 1. Introduction

Cancer is one of the main causes of mortality in the world. Acute lymphoblastic leukemia is the most common malignant type in children in such a way that it accounts for 75% of the cases of leukemia. This type of leukemia in boys is twice as much as the girls accounting for around 30% of all types of malignant cases in the childhood period (Baraz, Mohammadi, & Broumand, 2006).

In the last decade, improvements in health-care methods have led to an increasing rate of survival in children afflicted with leukemia. Fortunately, against the one-percent annual increase in the rate of cancer breakout, the successful treatment of this disease increases 0/25 per cent annually (Tsay, 2003).

The parents of the children afflicted with cancer undergo psychological problems such as anxiety, stress, depression, and so on. They as the first and most important care-givers of the children, are confronted with different problems such as the symptoms of a disease, drugs and their side effects, providing non-pharmacological measures, financial, family and social problems (Hanoch, 1999).

Quality of life is a complex issue. The concepts used for understanding quality of life include satisfaction and dissatisfaction, quality of life, happiness, unhappiness, life experience, and factors as comfort, performance situation, socio-economic situation, independence and environmental conditions. Quality of life is the best criterion for measuring a person’s capacity for a successful adjustment with the existing challenges in the real world (Shoaa Kazemi & Momeni, 2010).

Long-term vulnerability to cancer in childhood will have a negative effect on the parents’ quality of life (Litzemank, 2011). A decreasing degree in the quality of life and a breakup of their life patterns will lead to an irregular pursuit of the disease and it is not being treated (6). Presenting a clear definition for quality of life is a difficult task; however, it can be said that the way to “quality of life” is to help people put their potential capacities, by way of choosing the best way, into action (Shoaa Kazemi & Momeni, 2010).

Therefore, designing and executing instructional programs with a great emphasis on improving the quality of life of the parents of children afflicted with leukemia is of special importance which can, on the one hand, by boosting the sense of independence, increase their moral and power and the merit of the parents when facing problems arising from the disease and, on the other hand, by instructing issues as regards leukemia and the way to its treatment, the effect of the disease on other organs, the side effects of chemotherapy and the way to its control and restraint, the methods of establishing rapport with the patient child, and the way to reducing existing problems, it can affect parents’ lives. Through this procedure, their quality of life can be ameliorated.

The effectiveness of the programs of health instruction depends, to a great extent, on the appropriate use of health training. Choosing suitable instructional methods depends on many factors such as the type and the intensity of the disease, the type of the help-seekers, existing facilities, and similar cases. In the pediatrics department, concerning leukemia, most of the instructional programs offered to parents are in the form of a pamphlet or very short individual instruction. In this method, oral presentation is used for explaining the materials and the parents are inactive receptor learners and their personal differences are not attended to. On the one hand, mostly, because of the insufficiency of the staff, the quality of the instructions is not so desirable. Instruction through group discussion is an active and help-seeker-oriented instructional method in which learners, using discussion, participate in active instructional activities and they are given the opportunity to share their comments and experiences with others. Teaching on a group discussion method basis, grows the power of criticism in learners (Safari, Yazdanpanah, Ghafarian, & Yazdanpanah, 2011). Teaching through learning groups are based on teamwork and parameters which embrace motivation arising from synergy and the domination of teamwork over individualism, the acquisition of the members of the group from each other, achieving higher levels of mental processes, increasing positive feeling compared with other learners, reinforcement of self-esteem and an increase in the general social skills (Joyce, Weil, & Calhoun, 2001). Instruction and learning on a group discussion method basis, irrespective of instructional dimensions and having enough chance to analyze the points in discussions, has undeniable effects for promoting communication and social culture of the individuals. This method is valuable in ameliorating communicative skills, developing self-confidence by discussing about a subject, improvement of power in conveying and making a point clear, reinforcement of the power to listen, observing the reaction of others to what is said, objection without resistance and partiality, free expression of comments and posing questions mutually which are sometimes the starting point of an investigation. In general, discussion, thinking, understanding, learning and remembering help all the interested people take advantage of this method (Barrass, 2002).

So far, several studies have attended to the investigation of the effect of group discussion instruction method on awareness, the attitude and the performance of help-seekers afflicted with different diseases. A study by Ghavam Nasiri et al. (2010), concerning the comparison of the effect of self-caring instruction on an individual and group basis on the quality of life of the patients under chemotherapy, showed that both methods led to an improvement in the quality of life and their difference was not significant and meaningful clinically.

Also, a study by Barim Nejad et al. (2012), concerning the effect of individual and group instruction on the pursuit of treatment and the rate of the emergence of side effects in patients consuming warfarin after changing the heart valve in Tehran, showed that group instruction has been more effective as opposed to individual instruction (Barimnejad, Assemi, Samiei, & Haghani, 2012). Shirazi et al. (2010) attended to the investigation of self-caring instruction program through group discussion on the knowledge and performance of female juvenile afflicted with diabetes in Tehran (Shirazi, Anousheh, & Rajab, 2010). All these studies emphasize the necessity of executing instruction-oriented intervention for promoting the quality of life of the help-seekers afflicted with chronic diseases. Therefore, it seems that the parents of children afflicted with leukemia can have an improved quality of life through learner-oriented instruction.

In Iran, no study has been carried out on the investigation of the effect of this instructional method on the quality of life of the parents of the children afflicted with leukemia. Thus, the present study was carried out with the aim of investigating the effect of instruction on a group discussion method basis on the quality of life of the parents of the children afflicted with leukemia.

## 2. Material Studied

### 2.1 Setting and Sampling

This study is a semi-empirical study including two groups on a pre-and-post intervention basis. The persons under study were accessible samples out of the parents of the children afflicted with leukemia clients in oncology department of the pediatrics hospital of Bandar Abbas city in 2013. Although the sample of participant represents a convenience sample, sampling was done by census and the total of 50 individuals participated in the study. Based on a formula, the volume of the study comprised of 50 individuals qualified for the study who were inclined to take part in the study and were, randomly, placed in two groups consisting of 25 intervention and control groups. The criteria for entrance into the group were as follows:

1) The child should not have any other known chronic disease except leukemia.
2) The family should be of a nuclear type.
3) Other members of the family should not have either leukemia or any other known disease.
4) None of the units under investigation should have prior experience of cancer and should not have used any of the advisory systems.
5) They should be inclined to participate in the study.

Firstly, the researcher called on the pediatrics hospitals and obtained the permission from the management of hospital for carrying out the research. All the participants had, consciously and voluntarily, participated in the sampling and they were assured of the confidentiality of the information and it was stated that they could quit any time they desired. In the intervention group, four individuals were left out of the study for non-complete participation in the instructional sessions and five individuals in the control group for non-completion of the questionnaire. Finally, the analysis of the data was carried out on 41 individuals.

### 2.2 Instruments

For collecting the data, two questionnaires were used 1) a questionnaire concerning demographic information including questions as regards age, gender, level of education, the job of the parents, the number of children, and the period of time elapsed from the commencement of treatment 2) a short form of SF36 questionnaire for determining the parents’ quality of life: this questionnaire evaluates health in two dimensions of “physical and mental”. In this questionnaire, the nearer the mean of scores to zero, the lower is the quality of life and the nearer the mean of scores to 100, the higher the quality of life. This questionnaire has a high degree of reliability and validity. So far, its short form has been repeatedly used in Iran. This instrument has been standardized and designed by Montazeri et al for the population of Iran (Khanjari, Oskouie, Eshaghian Dorche, & Haghani, 2013). A study by Taraghi et al in Mazandaran investigated its validity and the Cronbach’s Alpha coefficient was reported to be 77-90 (Shoaa Kazemi & Momeni, 2010). Also, Mo’tamed et al investigated the reliability of the questionnaire in Shiraz and the Cronbach’s Alpha coefficient was estimated to be 87% (Zarea, Baraz Pordanjani, Pedram, & Pakbaz, 2012).

The SF36 questionnaire short form had 36 questions on general health areas (6 questions), physical health (10 questions), mental health (5 questions), physical limitation (4 questions), mental limitation (3 questions), vitality (4 questions), social relations (2 questions) and pain (2 questions). Point-giving to different parameters of this questionnaire were in terms of the type of the question in the form of 1-6, 1-5, 1-3, and 2-1 and the maximum point obtained is equal to 100 being classified into five levels of “excellent” (80-100), very good (60-79), good (40-59), bad (20-39), and very bad (0-19). The questions 1, 2, 20, 22, 23, 33, 34, 35, 36 are rated on a five-level likert scale, the questions of 1, 2, 3, 4, 5, 6, 7, 8, 9, 10, 11, 12 on a three-level likert scale and the questions of 21, 22, 23, 24, 25, 26, 27, 28, 29, 30, 31 on a six-level likert scale.

After placing the parents in two groups, the individuals in the intervention group were called to take part in groups of 4-5 individuals. At the end, in the course of three phases of time, eight one-hour instructional sessions were held for each group by two experienced nurses. In the instructional sessions, in the first phase, some pieces of information were stated about anatomy, physiology, the body’s hematopoietic system, the disease of leukemia and the way of its treatment. In the sessions of the second and the third phase, the discussion revolved round chemotherapy, and its side effects, the way of controlling any of the side effects, palliative cares and controlling the pain, the way to nutrition, exercising and the way to communication and adjustment with these children and the importance of the parents’ quality of life in taking care of the diseased child.

Both groups completed the questionnaire related to the quality of life before the intervention and two months after the intervention.

### 2.4 Data Analysis

The information were analyzed using SPSS software, edition 19, and independent t test for comparing the mean of the scores of the test between intervention and control groups, the dependent t-test for comparing the mean of the scores in the pre-and-posttest, χ^2^ test for comparing the qualitative variables and the Pearson correlation coefficient for the connecting the quantitative variables of each group at a significant level of 0/05.

## 3. Results

### 3.1 Sociodemographic Characteristics

The analysis of data was carried out on 41 patients (18% fall). The average age of the patients was 33.8 in the intervention group and 35.5 in the control group and 73.2 percents of the participants were mothers of children afflicted with leukemia. 41.5 percents were non-literate and 70 percents were employed. The average period for the affliction to the disease was 2.7 years. 53.7 percents of the participants reported having a weak economic situation.

The χ^2^ test showed no significant difference between the control and intervention group demographically.

### 3.2 Knowledge in Quality of Life

The dependent statistical t-test showed a significant difference between several dimensions of the quality of life in the post-stage of intervention compared to the previous stage (Table 1).

**Table 1:** Comparing the mean of scores in 8 areas of quality of life of mothers of children afflicted with leukemia before and after intervention in two groups of control and intervention

variabe		Pre intervention		Post intervention			p-value
	
		Mean	SD	Mean	SD	t	
Physical functioning	intervention	67.8	28.9	69.4	64.4	-0.5	0.5
	control	67.8	23.1	25.3	19.6	1.4	0.16
Role Limits: physical	intervention	60.1	44	67.5	55.9	-1.8	0.07
	control	60.2	42.2	36.3	42.2	2	0.05
Bodily pain	intervention	58.7	24.6	68.1	56.5	-2.6	0.01
	control	60.6	23.2	36.3	42.2	1.4	0.16
General health	intervention	41.3	25	60.5	20.7	-6.6	<0.001
	control	41.5	25.1	40.1	23.4	1.4	0.16
Emotional health	intervention	39.8	20.3	60	37.3	-4.8	<0.001
	control	40.8	19.2	10.4	18.3	1.2	0.2
Role limits: emotional	intervention	37.5	24.3	65.6	35.4	-5.9	<0.001
	control	36.1	23.3	15.3	22	0.2	0.8
Social functioning	intervention	32.1	35.4	80.9	29.5	-5.8	<0.001
	control	31	34.4	20.9	33.6	2	0.05
Vitality	intervention	41.9	16.4	62.02	11.8	-5.1	<0.001
	control	41.2	16.3	40.6	15.5	0.9	0.3

The independent t-test showed no significant difference in the score of different dimensions of quality of life of the two instruction groups through group discussion and instruction based on the routines of the department in the pre-stage of intervention (p>0.0.5); however, after the intervention, the score of several dimensions had a significant difference in both groups (p<0.05) (Table 2).

**Table 2:** Comparing the mean of scores in 8 areas of quality of life of mothers of children afflicted with leukemia after intervention in two groups of control and intervention

variable	Intervention group		Control group		t	p-value
	
	mean	SD	mean	SD		
Physical functioning	69.4	25.3	64.4	19.6	0.6	0.4
Role Limits: physical	67.5	36.3	55.9	42.2	0.9	0.3
Physical pain	68.1	19.3	56.5	22.4	1.7	0.08
General health perceptions	60.5	20.7	40.1	23.4	2.9	0.005
Emotional health	60	10.4	37.3	18.3	4.8	<0.001
Role Limits: emotional	65.6	15.3	35.4	22	5.1	<0.001
Social functioning	80.9	20.9	49.5	33.6	5.9	<0.001
Vitality	62	11.8	40	15.5	4.9	<0.001

**Table 3:** Comparing of overall dimensions SF-36 questionnaire mean scores of mothers of children with leukemia before and after intervention

Variable		Pre intervention		Post intervention		t	P-value
	
		Mean	SD	mean	SD		
Physical health	Intervention	46.7	12.5	48.8	12.2	-2.9	0.009
	control	45.7	8.6	43.1	7.9	4.5	<0.001

Mental health	Intervention	25.3	5	42.1	5.8	-7.7	<0.001
	control	32.7	6.5	31.5	7	0.8	0.3

No significant difference was observed between the quality of life in the areas of physical and mental health and variables such as age, and occupation while the mental and physical health had a significant difference with the variable of family income rate (p<0.05). Also, the Pearson correlation coefficient showed no significant relation between the period of affliction to the disease and the score of quality of life in two dimensions of mental and physical health before and after the intervention.

## 4. Discussion

The results of the present study showed that training the way to caring a child afflicted with leukemia through group discussion can improve the quality of the life of the parents. The results of the study carried out from the rate of knowledge and skill of parents about the way of caring a child afflicted with leukemia, palliative care, controlling the pain and controlling the side effects chemotherapy before the beginning of the study were indicative of the lack of sufficient information in many cases. Also, the results of the study carried out before the commencement of intervention showed that the parents of children afflicted with leukemia have a low quality of life. Many studies have examined the quality of life of the parents of children afflicted with chronic diseases and have attend to the undesirable effect arising from the affliction of the children with these diseases on the quality of life and physical and mental health of the parents and care-givers. The results of these studies are in line with the present study and have reported the score of the quality of life to be low such as a study by Arafa in Egypt (2003) or LitzManek in the U.S (2011), Klasson in Canada (2008), Yamazaki in Japan (2005), and Fiddik in Germany (2013), Khanjari et al. (2013) and Zare et al. (2012) in Iran, this issue has been emphasized that taking care of a child afflicted with chronic diseases or diseases hard to treat such as leukemia can, whether bodily or mentally, have negative effects on the parents and will lead to a decrease in the quality of life. Many studies have attended to the investigation of the effect of instructing child-caring program on the quality of life of the children afflicted with leukemia or other chronic diseases such as a study by Hashemi et al. (2011), vojdani et al. (2011), Allah Yari et al. (2006), Agha Seyyed Mirza et al. (2013), Farrokhnia et al. (2011) all showed that one can improve the quality of life of the children by instructing the parents concerning the way of child-caring. In the present study, by comparing the average scores of the quality of life of the parents, before instruction and two months after instruction, improvement was observed in six areas including bodily pain, general health, and emotional health, limitation in playing a role due to emotional problems, social functioning and vitality. The parents should be paid attention to as the most important child care-givers afflicted with diseases hard to treat. Taking care of the diseased child is endangered as the quality of life lowers. Thus, considering the instructional programs for improving the parents’ quality of life, ultimately, can lead to a better care and an improvement in their quality of life. Compared with other chronic diseases of children, very few studies have considered the investigation of the effects of instruction intervention on the quality of life of children’s care-givers afflicted with leukemia. Maybe we can justify the causes of this difference in this way that, in the previous decades, many advances have been made in treating the children afflicted with leukemia and these children can continue their lives for several years while they would die as a result of this disease. Alaee et al. (2010) attended to intervention investigation, in a review study entitled “nursing intervention for improving the adjustment of the parents of children afflicted with cancer”. Their intervention causes a reduction in stress, improved performance and adjustment of parents and children. According to the educational intervention have been mentioned in this study, there are some information as regards the side effects of the disease and the way of their controlling which cause the reduction of anxiety and the problems of the families and children and the acceptance of treatment and a sense of control are improved. The parents want to dominate the disease by exact comprehension of problems. Acquisition of knowledge by them will cause the control of feeling and avoiding frustration (Last & Grootenhuis, 1997). As it was said, concerning the effect of instructing care program on the quality of life of the parents or the care-givers of children afflicted with other chronic diseases, several studies have been carried out. One can refer to the studies conducted by Hashemi et al. (2011); edraki et al. (2014); Khanjani et al. (2009); Baharloo et al. (2002); and Jalili et al. (2013). The results of these studies, as the present one, are indicative of the emergence of positive effects in care-givers after instructional intervention. However, in any of these studies, improving the quality of life has occurred in different dimensions which can be a result of difference in the type of the chronic disease, the way of treating a patient, difference in the population under study, difference in the time and the intensity of care or even difference in the measurable instrument. In the present intervention, the created changes in the area of physical health did not become significant and meaningful. The cause can be the shortness of instruction period. Also, probably, the parents of the children afflicted with leukemia are much more mentally affected at the time of diagnosis.

Limitations of this study include its small sample size and the fact that the mothers of children afflicted with leukemia were enrolled in one nursing program. Future research could focus on larger samples from different care-givers programs. It may also be interesting to examine care-givers’ experiences in different practice settings, such as different pediatrics Hospitals.

## 5. Conclusion

Considering the importance of parents in taking care of the child afflicted with chronic diseases such as leukemia, heeding their health, identifying the obstacles, and attempt in improving their physical and mental health are of special importance. Instruction of care programs to parents can be a positive step for enabling them to manage the problems of the child and a more principled care which, ultimately, can lead to an improved quality of life of the parents and children.
